# Carboplatin sensitivity in epithelial ovarian cancer cell lines: The impact of model systems

**DOI:** 10.1371/journal.pone.0244549

**Published:** 2020-12-31

**Authors:** Bishnubrata Patra, Muhammad Abdul Lateef, Melica Nourmoussavi Brodeur, Hubert Fleury, Euridice Carmona, Benjamin Péant, Diane Provencher, Anne-Marie Mes-Masson, Thomas Gervais

**Affiliations:** 1 Department of Engineering Physics and Institute of Biomedical Engineering, École Polytechnique de Montréal, Montréal, QC, Canada; 2 Centre de Recherche du Centre Hospitalier de l’Université de Montréal (CRCHUM) and Institut du Cancer de Montréal, Montréal, QC, Canada; 3 Division of Gynecologic Oncology, Université de Montréal, Montréal, QC, Canada; 4 Department of Medicine, Université de Montréal, Montréal, QC, Canada; Indiana University School of Medicine, UNITED STATES

## Abstract

Epithelial ovarian cancer (EOC) is the most lethal gynecologic malignancy in North America, underscoring the need for the development of new therapeutic strategies for the management of this disease. Although many drugs are pre-clinically tested every year, only a few are selected to be evaluated in clinical trials, and only a small number of these are successfully incorporated into standard care. Inaccuracies with the initial *in vitro* drug testing may be responsible for some of these failures. Drug testing is often performed using 2D monolayer cultures or 3D spheroid models. Here, we investigate the impact that these different *in vitro* models have on the carboplatin response of four EOC cell lines, and in particular how different 3D models (polydimethylsiloxane-based microfluidic chips and ultra low attachment plates) influence drug sensitivity within the same cell line. Our results show that carboplatin responses were observed in both the 3D spheroid models tested using apoptosis/cell death markers by flow cytometry. Contrary to previously reported observations, these were not associated with a significant decrease in spheroid size. For the majority of the EOC cell lines (3 out of 4) a similar carboplatin response was observed when comparing both spheroid methods. Interestingly, two cell lines classified as resistant to carboplatin in 2D cultures became sensitive in the 3D models, and one sensitive cell line in 2D culture showed resistance in 3D spheroids. Our results highlight the challenges of choosing the appropriate pre-clinical models for drug testing.

## Introduction

Epithelial ovarian cancer (EOC) is the 5^th^ cause of cancer-related deaths in North American women with a 5-year survival of 45% [1]. The standard front-line treatment for EOC includes cytoreductive surgery and treatment with platinum DNA alkylating agents such as carboplatin or cisplatin combined with the anti-microtubule drug paclitaxel [2]. Although initial response rates are high (>70%), the disease eventually recurs in most patients, who will then develop chemoresistance [2–4]. To improve EOC survival, new therapies and their combinations are under investigation, and preclinical models are used to test their efficacy prior to eventual human trials. Because of time and cost efficiency, these models are often in the form of 2D or 3D *in vitro* assays. However, optimizing and translating the results seen in 2D *in vitro* models to an *in vivo* response is challenging, as these models do not take into account important tumor characteristics such as hypoxia, drug transport restriction, extra cellular matrix (ECM), anchorage-independent growth as well as interactions with other cell types in the tumor, including stroma/fibroblast and immune cells [5–8]. Alternatively, 3D spheroid models have been proposed as an attractive alternative as they are thought to more closely resemble the tumor tissue architecture [9–11].

There are several methods to prepare 3D spheroids from cell lines (reviewed elsewhere [11, 12]) including hanging-droplets [13–16], rotating wall vessel cultures [14, 17], and ultra-low attachment (ULA) 96-well round-bottom plates [11, 18, 19]. In ovarian cancer, several groups have described the capacity of EOC cell lines to form spheroids by different methodologies [20–26], including our work using hanging droplets [16, 27–29]. These different techniques each have their own challenges. On one hand, hanging-droplet spheroids take longer to form, are difficult to manipulate for spheroid transfer steps, and they present a challenge for experiments requiring medium changes [30, 31] or treatments. On the other hand, spheroids formed in ULA plates can be readily used in high-throughput assays [18, 19] but many cell lines do not form spheroids under these conditions. Considering the importance of the tissue microenvironment in the establishment of 3D models, components of the ECM have been used to assist spheroid formations [11]. Matrigel, consisting of collagen type IV, perlecan/heparin sulfate proteoglycan 2 and laminin, is one of the most commonly used ECM mimics [11, 32] and has been shown to improve spheroid formation in ULA plates for cell lines that otherwise would not have the capacity to aggregate [18, 19]. More recently, many bioengineered microfluidic devices have been developed and shown to be effective in forming spheroids [33–39]. In addition, the use of these microfluidic devices has gained popularity as a 3D model system for drug screening [12, 33–44]. In ovarian cancer, our group has been a pioneer in the application of this technology to evaluate drug response in EOC cell lines [37, 38, 41].

**Fig 1:**
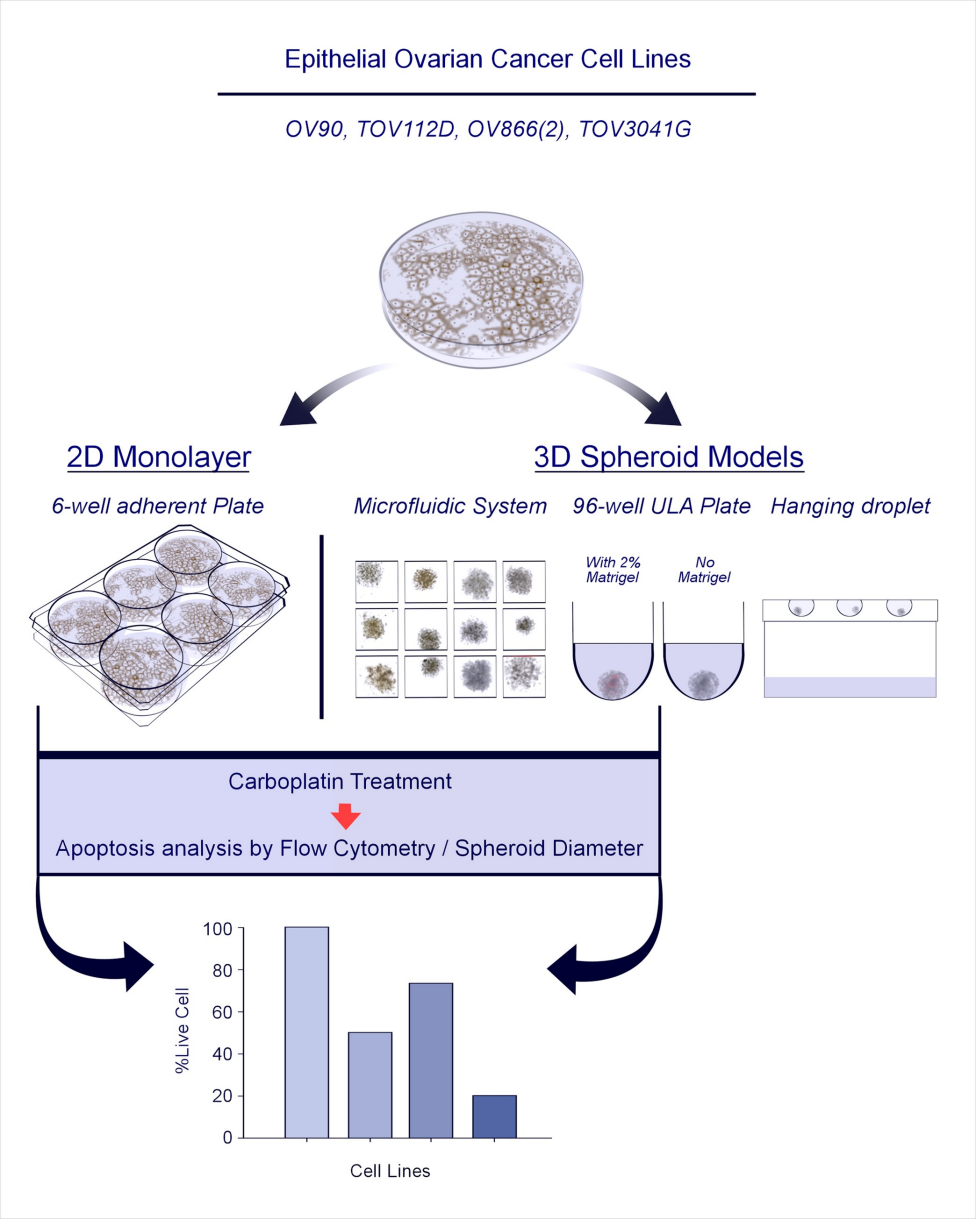
Schematic illustration of the study strategy. For each cell line used, 3D spheroid formation was evaluated by four different methods, i.e., PDMS-based microfluidic systems, ultra-low attachment (ULA) plates in the presence or not of 2% Matrigel, and hanging droplets. The two most effective 3D spheroid methods (microfluidic chips and Matrigel-assisted ULA plates) were used to define carboplatin sensitivity of each cell line that was then compared to responses in the 2D monolayer assay.

Although several studies have compared the chemosensitivity of ovarian cancer cell lines grown as monolayers with 3D-spheroid cultures [18, 41, 45, 46], little is known about the impact that different 3D spheroid model systems have on their sensitivity. In the present study, we investigated the ability of four different EOC cell lines to form 3D spheroids following different methodologies (hanging-droplets, ULA plates and microfluidic chips), and their carboplatin sensitivity in monolayer culture versus spheroids formed within microfluidic devices or round-bottom ULA plates (the latter using Matrigel as an ECM) (Fig 1). Response was measured by flow cytometry for apoptosis/cell death and spheroid size measurement. Our results show that spheroid formation of EOC cell lines is significantly faster and more uniform in polydimethylsiloxane (PDMS) microfluidic devices and Matrigel-assisted ULA plates than in hanging-droplets or ULA plates without Matrigel. Carboplatin responses were observed in both 3D spheroid models using flow cytometric analysis, but no significant decrease in spheroid size was detected. For the majority of the EOC cell lines (3 out of 4) a similar response to carboplatin treatment was observed by both spheroid methods. Interestingly, two cell lines classified as resistant to carboplatin in 2D cultures responded as sensitive in 3D models, and one sensitive cell line in 2D culture showed resistance in the 3D spheroids. Our results highlight the challenges in choosing appropriate pre-clinical models for empirical drug testing.

## Materials and methods

### Cell lines

Four different human EOC cell lines were used in this study, TOV3041G, TOV112D, OV90 and OV866(2) (all BRCA1/2 wild-type). Three cell lines [TOV112D, OV90, OV866(2)] are classified as resistant to carboplatin therapy in 2D culture as measured by clonogenic assay, and one (TOV3041G) is classified as sensitive (Table 1). These cell lines were derived in our laboratory from patient tumor (TOV) or ascites (OV) [27, 47] samples. All cell lines were authenticated at the beginning of this study using STR profiling by the McGill University Genome Center (Montreal, Canada). Cells were cultured in complete OSE medium (Wisent, QC, Canada) supplemented with 10% fetal bovine serum (Wisent), 2.5 μg/mL of amphotericin B (Wisent) and 50 μg/mL of gentamicin (Wisent). Cells were maintained in 100 mm petri dishes with 5% CO_2_ at 37°C.

**Table 1:** Carboplatin sensitivity (IC_50_ by clonogenic assay) of the EOC cell lines used in this study.

	Carboplatin IC_50_ (μM)	Reference
TOV3041G	5.2 ± 1.1	Fleury et al. 2015
TOV112D	13.4	Letourneau et al. 2012
OV90	31.8 ± 5.4	This study
OV866(2)	32.1 ± 7.1	Fleury et al. 2015

### Clonogenic survival assays

The IC_50_’s for carboplatin, as determined by clonogenic survival assay, have previously been reported for TOV3041G, TOV112D, and OV866(2) [27, 28]. Carboplatin sensitivity for the OV90 cell line was determined in this study using the same clonogenic assay [27]. Briefly, cells were seeded in a 6-well plate at a density of 750 cells/well that allowed the formation of individual colonies. After seeding, cells were allowed to adhere for 16 hours in a 37°C, 5% CO_2_ incubator after which the medium was removed and replaced with OSE complete medium containing carboplatin (0–100μM) (Hospira Healthcare Corporation, Saint-Laurent, QC). Cells were incubated with the drug for 24 hours. The drug was then removed and OSE complete medium was added to each well. When colonies became visible at a 2X magnification plates were fixed with cold methanol and stained with a solution of 0.5% blue methylene (Sigma–Aldrich Inc., St. Louis, MO) in 50% methanol. Colonies were counted under a stereomicroscope and reported as percent of control. IC_50_ values were determined using Graph Pad Prism 5 software (GraphPad Software Inc., San Diego, CA). Each individual experiment was performed in triplicate and repeated three times.

### Spheroid formation

**Fig 2:**
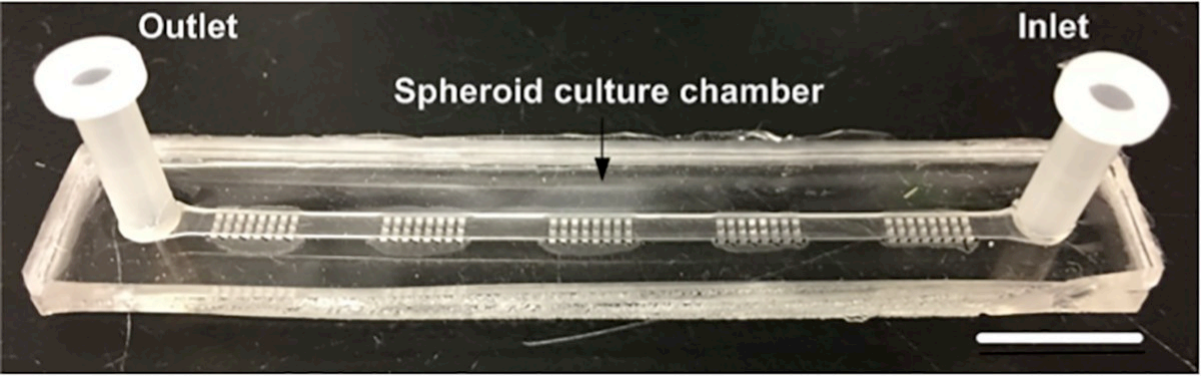
Design of PDMS microfluidic device developed for the culture of 3D spheroids and drug testing. Each chip is comprised of five sections of 24 wells, containing 120 wells. Each well has a dimension of 500×500×500 μm^3^. The scale bar is 1 cm.

Spheroids were formed using three different methods: hanging-droplet, PDMS-based microfluidic chips and ULA 96-well plates (with and without Matrigel). Hanging-droplet spheroids were generated as previously described [16]. Briefly, 16 μL droplets containing 4000 cells each were placed on the inside of the lid of a 150 mm cell culture plate. The lid was then gently inverted and replaced on the base of the plate containing sterile PBS for humidification and the plate was put in the incubator with the droplets hanging. Spheroids were allowed to grow for up to 6 days. For the microfluidic method, each device contained 120 spheroid culture chambers, with one inlet and one outlet (Fig 2, see fabrication section below). A cell suspension of 1 x 10^6^ cells in 1 mL of complete OSE medium was used. The device was seeded with 100 μL of this suspension in the inlet, and the same amount of medium was removed from the outlet. This process was repeated by seeding in the outlet and removing medium from the inlet and performed five times in each direction (total 10 times, using all of the 1 x 10^6^ cells) to allow uniform distribution of cells across the wells. Spheroids were allowed to form over 48 hours. Finally, spheroids were also prepared using 96-well round-bottom ULA plates (Corning 4515). Wells were seeded with 2000 cells in 100 μL of cooled complete OSE medium with or without 2% Matrigel (BD, Bioscience, Canada). This was done for each cell line and condition. Spheroids were allowed to form over 48 hours.

### Microfluidic device fabrication

The PDMS microfluidic device used in this work is a modification of a previously described device [35]. It was made of PDMS and used the basic principle of gravity trapping [34, 41, 48]. The device layout was developed using CATIA (Dassault Systèmes, France) and the mold was carved out of polymethyl methacrylate. Using this mold, our device was made of two bonded layers of PDMS (Fig 2); 1) the bottom later contains five segments of spheroid culture chambers, each of which contains 24 wells of 500μm x 500μm x 500μm, and 2) the top layer contains a straight channel with a height of 500μm covering the entirety of the spheroid culture chambers with 3 mm holes for the inlet and outlet at the ends of the straight channel. The device was bonded using an upright microscope to align the layers after 30 sec of atmospheric plasma surface treatment. After bonding, the device was put in an oven at 80° C for two hours. The dimension of the device was 7.5 cm long x 1 cm wide with of 120 spheroid culture chambers (5 blocks of 24 chambers each). Nine devices could be culture at a time in an incubator using a simple pipet tip box. Prior to use, the devices were treated with 10 mg/ml Pluronic (Sigma-Aldrich, St Louis, USA) as previously described [48], to make the PDMS surface less adherent to cells. This treatment also prevents chemo-absorption by the PDMS [49].

### Carboplatin treatment

Previously, we have shown that the carboplatin IC_50_ for spheroids from a specific EOC cell line (TOV112D) is approximately 10x the corresponding monolayer IC_50_ value [41]. Therefore, as the most resistant cell line in this study has an IC_50_ of 30μM (Table 1), we treated the spheroids formed in the PDMS-based microfluidic systems and in the ULA plates with 300 μM carboplatin. Moreover, in our earlier pilot studies using concentrations 10 times lower (30 μM) and 10 times higher (3000 μM), no differential results among the cell lines were observed, as they all survived at the lower dose and all died at the higher. Interestingly, the 300 μM carboplatin concentration is similar to that found in the plasma of ovarian cancer patients undergoing chemotherapy [48]. Spheroids formed 48 hours after cell seeding were treated with fresh medium (controls) or fresh medium containing 300 μM carboplatin (Hospira Healthcare Corporation) for a period of 48 hours. Carboplatin treatment was also performed on monolayer cultures prepared with 5,000 cells/well of each cell line seeded into 6-well plates. Consistent with the method for spheroids, 48 hours after inoculation, the cultures were treated with fresh medium (controls) or fresh medium containing 300 μM carboplatin and cultures were incubated for 48 hours. After treatment samples were immediately used in subsequent analyses.

### Microscopy and imaging

Microscopy images were taken using an inverted microscope (Nikon TS100) at 10X or 4X magnification two and four days after seeding as well as 48 hours after treatment prior to flow cytometry analysis. PDMS microfluidic devices were put into a 100 mm petri dish for imaging purposes to maintain sterile conditions. Size estimation was done using ImageJ software (Version 1.49, National Institute of Health, Bethesda, MD).

### Flow cytometry analysis

After carboplatin treatment, monolayer cells from each well of the 6-well plates were harvested by trypsin-EDTA (0.05%) as a single cell suspension. For the spheroids treated in the ULA plates, 10 were collected, pooled and dissociated with trypsin-EDTA (0.05%) for 5 minutes to obtain single-cell suspensions. For those treated in the PDMS-based microfluidic devices, 48 were pooled and dissociated as above. Cells were then labelled with Annexin-V (3:100 dilution) and 7-Amino-ActinomycinD (7-AAD, 5:100 dilution) to detect apoptotic and dead cells, respectively (PE Annexin V apoptosis Detection Kit I, BD Biosciences). After a 15-minute incubation at RT (25°C) in the dark, the stained cells were analyzed by flow cytometry (LSR-Fortessa, BD Biosciences), within 2 hours of staining. The data collected from each acquisition was analyzed using the FlowJo software (FlowJo, LLC, Ashland, USA). After excluding cell debris, viable cells were selected based on the absence of 7-AAD and/or Annexin-V markers (see example on S1 Fig).

### Statistical analysis

Values are expressed as the mean ± SEM (standard error of the mean), derived from at least three independent experiments. Single comparisons between two groups were determined by Student’s t‐test (paired, two-tailed). Comparisons between multiple groups were determined by Tukey ANOVA multi comparison test. *P* values < 0.05 were considered significant. All statistical analyses were done using GraphPad Prism 6 software (GraphPad Software Inc., San Diego, CA).

## Results

### Comparison of spheroid formation using different methods

**Fig 3:**
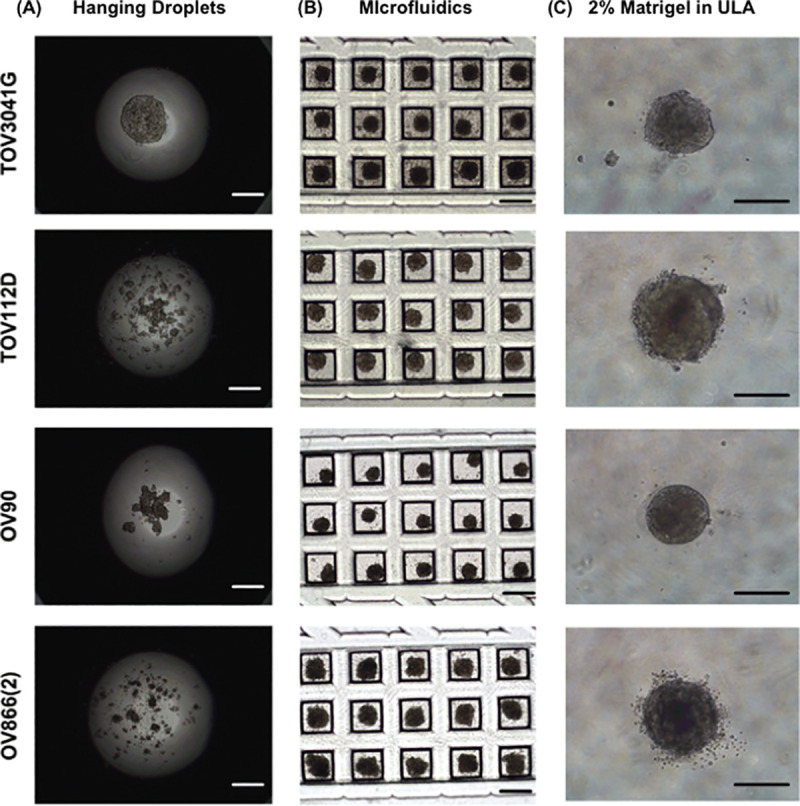
Capacity of EOC cell lines to form spheroids in three different culture conditions. Representative microscopic bright field images of TOV3041G, TOV112D, OV90 and OV866(2) cells cultured 48 hours in hanging droplets (A), within the custom PDMS-based microfluidic chips, (B) and in 96-well round-bottom ULA plates with 2% Matrigel (C). The scale bar is 500 μm.

In order to compare carboplatin sensitivity of different EOC cell lines in 2D monolayers and in 3D spheroids obtained by different methods, we first evaluated the efficiency of each 3D technique (hanging-droplets, ULA plates, and PDMS-based microfluidic chips) to form spheroids using four EOC cell lines [TOV3041G, TOV112D, OV90 and OV866(2)]. Fig 3A shows the hanging-droplet cultures at day 2 with the four EOC cell lines. Only TOV3041G formed spheroids within 48 hours. Careful observation showed that, at this time point, TOV112D and OV90 had started to form multiple smaller spheroids. However, OV866(2) had not formed spheroids at all. Culturing the same cell lines up to day 4 and day 6, we observed that the smaller spheroids of TOV112D and OV90 had fused together to form larger spheroids (S2 Fig). However, OV866(2) did not show any sign of spheroid formation. Fig 3B shows bright field images of 3D cultures at day 2 using the PDMS-based microfluidic device. All cell lines formed spheroids in the microfluidic device within 48 hours. Fig 3C shows the bright field images of Matrigel-assisted spheroids using ULA wells at 48 hours. Again, all the cell lines formed spheroid within 48 hours. However, less compact spheroids were obtained when EOC cell lines were grown in the ULA plates in the absence of Matrigel (S3 Fig). Therefore, carboplatin sensitivity was evaluated in 3D spheroids formed in the PDMS microfluidic device and the Matrigel-assisted ULA plate since the latter is widely used for drug sensitivity assays [11, 18, 19, 45, 50].

### Carboplatin sensitivity of EOC cell line spheroids obtained by two different methods

**Fig 4:**
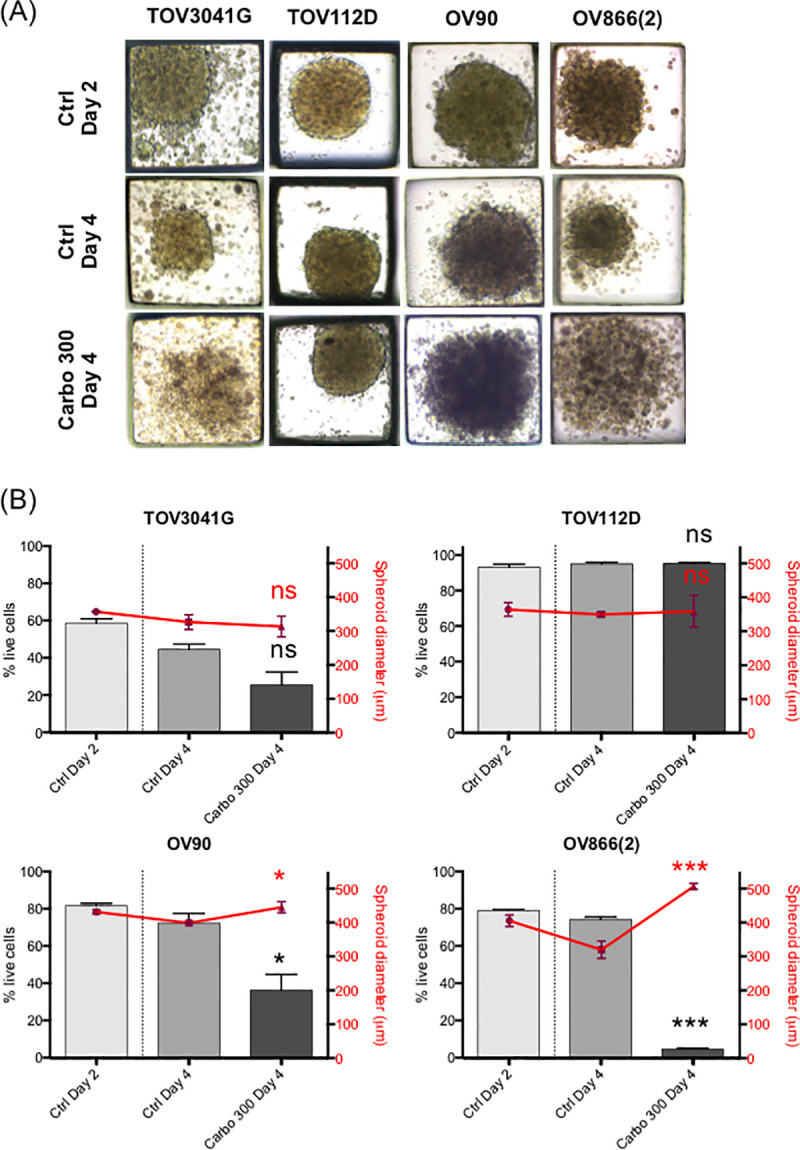
Carboplatin response of EOC spheroids cultured in PDMS microfluidic devices. 3D spheroids of four EOC cell lines [TOV3041G, TOV112D, OV90, OV866(2)] were formed and cultured in microfluidic devices for 2 and 4 days (Ctrl Day 2 and Ctrl Day 4) or treated at day 2 with 300 μM of carboplatin and assessed at day 4 (Carbo 300 Day 4). A) Representative microscopic bright field images at 10X magnification. B) Comparison of carboplatin treatment on EOC cell survival (grey bars) and spheroid diameters (red points). 48 spheroids were analyzed (in duplicate) from each set of experiment readouts. Error bars indicate the standard errors of the mean (SEM) of three independent experiments. Statistical analyses: Ctrl Day 4 vs Carbo 300 Day 4, * p<0.05; *** p<0.0005; ns, non-significant (Student’s t-test).

**Fig 5:**
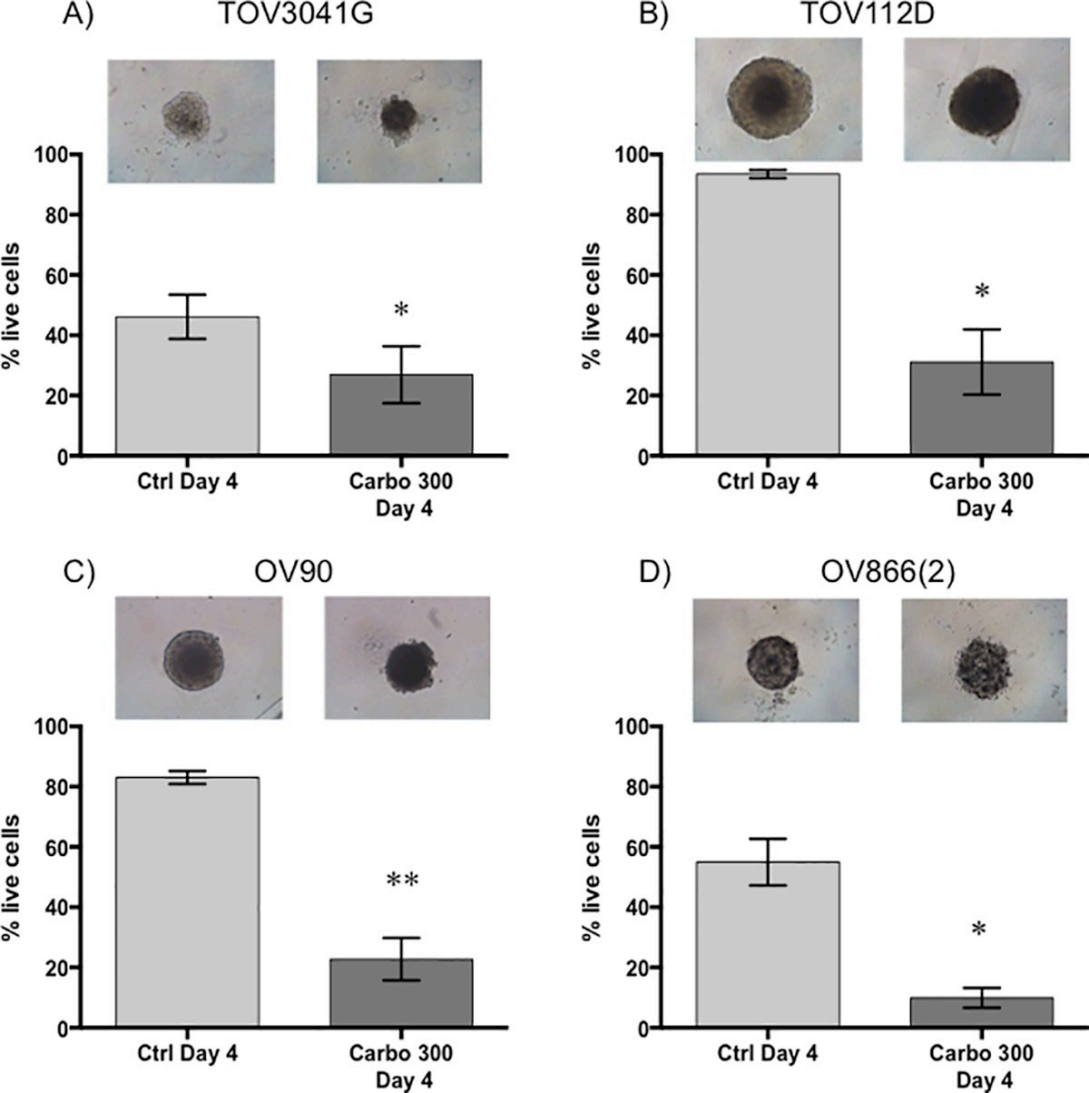
Carboplatin response of EOC spheroids cultured with 2% matrigel in ultra-low attachment wells. 3D spheroids of TOV3041G (A), TOV112D (B), OV90 (C) and OV866(2) (D) were formed and cultured in concave-bottom ULA plates in the presence of 2% Matrigel for 4 days (Ctrl Day 4) or treated at day 2 with 300 μM of carboplatin and assessed at day 4 (Carbo 300 Day 4). Top panels are representative microscopic bright field images of each condition. Bar graphs represent cell survival analysed by flow cytometry of control (light grey) and treated (dark grey) spheroids. Data are shown as the mean ± SEM of three independent experiments. * p<0.05; ** p<0.005 (Student’s t-test).

Sensitivity of the spheroids was evaluated at a concentration of 300 μM carboplatin, a concentration similar to that found in the plasma of ovarian cancer patients undergoing chemotherapy [48]. This concentration is in range of what has been used in other studies (100 μM, 330 μM) to evaluate platinum sensitivity of ovarian cancer spheroids [18, 46]. It is also 10 times higher than the 2D IC_50_ (by clonogenic assay) of the most resistant cell lines OV90 and OV866(2) (see Table 1). Then, the effect of carboplatin on our EOC cell lines was evaluated by measuring the size of the spheroids, as is commonly used in the literature [18, 19, 45, 51], and by measuring live/apoptotic/dead cells by flow cytometry using Annexin V/7-AAD staining of the dissociated spheroids. For the spheroids grown in the PDMS-based microfluidic chips, we observed a significant decrease in the percentage of live cells following treatment with 300 μM carboplatin for the OV90 and OV866(2) spheroids, but not for the TOV3041G and TOV112D cells, the latter being the most resistant in this assay (Fig 4B). However, no significant decrease in the size of spheroids was observed, and on the contrary, sizes of the OV90 and OV866(2) spheroids were significantly increased, likely due to spheroid disaggregation (Fig 4A and 4B). For spheroids grown in the Matrigel-assisted ULA plates, our results showed that all four cell lines presented decreased viability after incubation with 300 μM carboplatin, including TOV112D, as assessed by Annexin V/7-AAD staining and flow cytometry, but we did not notice a change in size when visually inspecting these spheroids (Fig 5 inserts). In the Matrigel-assisted ULA plates the highest resistance to carboplatin was observed in the TOV3041G cell line. Of note, sizes of non-treated spheroids were less than 500 μm in diameter in days 2 and 4 in both methods (Figs 3–5), which is important to avoid extensive internal necrotic core.

### Comparative carboplatin response of 3D spheroids with that of 2D monolayers

**Fig 6:**
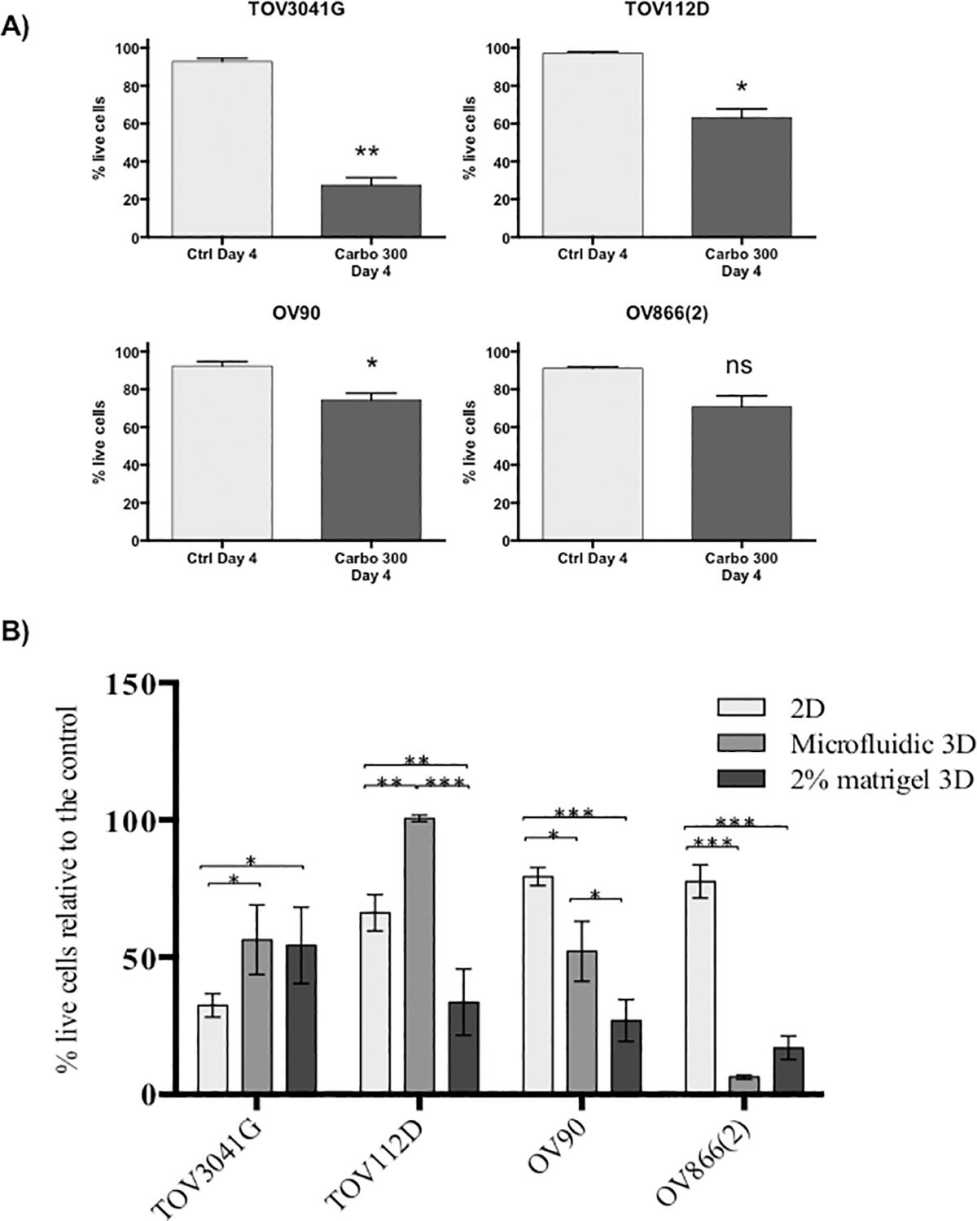
Comparison of carboplatin response between 2D-monolayers and two different 3D spheroid models. **A)** TOV3041G, TOV112D, OV90 and OV866(2) were culture as 2D monolayers for 4 days without treatment (Ctrl Day 4) or treated at day 2 with 300 μM of carboplatin and assessed at day 4 (Carbo 300 Day 4). Bar graphs represent cell survival analysed by flow cytometry of control (light grey) and treated (dark grey). Data are shown as the mean ± SEM of three independent experiments * p<0.05; ** p<0.005; ns, non-significant (Student’s t-test) **B)** 2D-monolayer carboplatin sensitivity was compared to that of 3D spheroid cultures. Each treatment response was normalized relative to the percentage of live cells in comparison to the appropriate control. * p-value <0.05; ** p-value <0.005; *** p-value <0.0005; Turkey ANOVA multi comparison test of three independent experiments.

The carboplatin sensitivity of cell lines in 2D culture was originally obtained using the clonogenic assay (Table 1), where cells are plated at a low dilution in order to obtain colonies from single cells. However, in our 3D spheroid assays (both methods), a large number of cells are used to form the spheroids and carboplatin sensitivity is determined by the percentage of residual live cells. In order to approximate analysis methods used for the 2D and 3D drug sensitivity assays, we treated cell monolayers at 80% confluence with carboplatin (300 μM), and evaluated cell viability by flow cytometry as for the spheroids. Sensitivity of 2D monolayers (Fig 6A) corresponded proportionately with that of the clonogenic assays where TOV3041G was the most sensitive and OV90 and OV866(2) the most resistant (Table 1).

When comparing the percentage of live cells by flow cytometry (normalized to controls) in the three methods tested, i.e. monolayer, spheroids from PDMS-based microfluidic devices and Matrigel-assisted spheroids, we observed that the spheroids of TOV3041G were significantly more resistant to carboplatin than in monolayer culture, and that the spheroids of OV90 and OV866(2) were significantly more sensitive than their respective monolayer cultures. This was independent of the spheroid formation method used (Fig 6B). However, when comparing both 3D cultures, OV90 spheroids formed in the microfluidic devices were significantly more resistant than those obtained using Matrigel-assisted ULA plates. However, both spheroid models were significantly more sensitive than the monolayer cultures. In contrast, spheroids of the TOV112D cell line formed in the microfluidic device were more resistant than the monolayer culture but were more sensitive when formed by the Matrigel-assisted technique. Table 2 summarizes this comparative study, in which a cell line ranking #1 has lower viability (more sensitivity) than the cell line ranking as #2, and this approach was further applied to determine rank order. When cell lines have similar percentage of cell survival, the same ranking is given to both of them. This classification shows a clear inversion of sensitivity from 2D to 3D for two of the four cell lines studied, in which the most sensitive becomes resistant to carboplatin (TOV3041G) and the most resistant becomes the most sensitive [OV866(2)].

**Table 2:** Relative carboplatin sensitivity of four EOC cell lines in three different culture conditions.

Cell line	2D culture model	3D culture model	
		PDMS Microfluidic devices	Ultra-low attachment wells and 2% Matrigel
TOV3041G	1	2	**3**
TOV112D	2	**3**	2
OV90	**3**	2	2
OV866(2)	**3**	1	1

## Discussion

In recent years, 3D cell culture models such as multi-cellular spheroids have become more widely used as pre-clinical models and are expected to bridge the gap between 2D and animal models. In the particular case of ovarian cancer, 3D spheroids are clinically relevant models since in advanced disease EOC cells can spread to the peritoneum as aggregates/spheroids [52, 53]. In the literature, several EOC cell lines have been studied by different spheroid generating methods, including hanging-droplets [16, 22, 27–29, 41, 54, 55], biomimetic hydrogels [23], plastic wells coated with matrix scaffolds [20, 46, 56], and more recently by microfluidics [33, 34, 37, 38, 42, 44] and commercially available round-bottom ULA plates [18, 45, 50, 57]. In the present study we first compared the ability of four EOC cell lines to form spheroids using these ULA 96-well plates (in the absence or presence of Matrigel) with that of hanging-droplets and PDMS-based microfluidic chips. For the latter, we used a recently developed [34, 37, 38] PDMS microfluidic device with a capacity of 120 spheroids per device. We showed that both Matrigel-assisted ULA plates and simple well-based PDMS microfluidic devices were the most robust and efficient methods to form spheroids. However, our microfluidic devices have the advantage of using much smaller volumes of media or drug, which is desirable when working with expensive compounds or extensive drug screenings.

We evaluated the carboplatin response of EOC cell lines grown as spheroids using these two methods and compared these to the respective monolayer cultures. Spheroid size is widely used as a measure of drug response in several studies, including those with EOC cells [18, 19, 45, 51], where decreased spheroid size has been interpreted as reflecting cell death [19]. In contrast, our results show that, independent of the method of spheroid formation used, no decrease in spheroid volume was observed. However, a significant increase in spheroid volume was observed for two cell lines [OV90 and OV866(2)] when using PDMS-based microfluidic devices, likely reflecting spheroid disaggregation due to cell death. Our findings indicate that spheroid size is not appropriate to be used as a measurement of carboplatin response, at least when using a short incubation time (like 48h of this study), as cell dissociation/disaggregation due to cell death/apoptosis would increase spheroid size. Therefore, the response of the spheroids and monolayers to the drug was also assessed by the broadly accepted method of flow cytometry through the analysis of apoptosis (Annexin V) and cell death (7-AAD). We observed decreased cell survival of spheroids after carboplatin treatment, with the highest level of cell death being observed in the two cell lines that showed increased spheroid volumes. Strikingly, in three of the four cell lines studied, the 3D sensitivity (independent of the spheroid formation method used) was inverted when compared with the corresponding 2D test, where two cell lines [OV90 and OV866(2)] classified as resistant in 2D cultures became sensitive in 3D models, and one sensitive cell line (TOV3041G) in 2D culture showed resistance in the 3D model. The latter result is more consistent with the literature where 3D spheroids are more resistant than 2D cultures [11, 18, 34, 41, 46, 58]. Although the exact signaling mechanism implicated in this resistance is still unclear, reports have attributed it to decreased compound access, reduced sensitivity in response to hypoxia, cells cycling more slowly, cell-cell contact influence, and an increased EMT phenotype [11, 12, 58].

In a recent report [18], a comparative analysis of cisplatin response of monolayer cultures and 3D spheroids of 16 EOC cell lines showed that the majority of the spheroids (13/16) were more resistant to cisplatin than the respective 2D cultures (much as we have observed with the TOV3041G cell line), whereas two cell lines were equally sensitive and one showed higher sensitivity in 3D spheroids than 2D monolayers, the latter similar to what we observed in two of our cell lines [OV9 and OV866(2)]. Another study using 11 EOC cell lines showed increased cisplatin resistance of 3D spheroids in seven cell lines, with no change for the other four [46]. It is possible too that the read-out methods used in these studies have underestimated spheroids cell death when compared to ours. In these articles, platinum sensitivity of ovarian cancer cell lines cultured as 3D spheroids have been mainly evaluated by metabolic assay (since it is a more high-throughput assay) that would overestimate cell survival, as early apoptotic or senescent cells are still metabolically active. We think that the cell death/apoptosis/cell viability FACS method used in our work, although less high-throughput, would more accurately estimate cell survival after carboplatin treatment in 3D spheroids. Nevertheless, all together, these findings imply that the assumption that spheroids are more resistant to chemotherapy than the monolayer cultures is not always accurate and may influence the choice of EOC models used to study chemoresistance. Previous publications have attempted to correlate molecular features (i.e. epithelial and mesenchymal protein biomarkers) with platinum sensitivity of ovarian cancer cell lines grown as 2D *vs* 3D [18, 46]. Based in these results, it is unlikely that these factors play a role in the sensitivity inversion of our cell lines.

When comparing the two spheroid models, we generally obtained similar carboplatin responses, except for the TOV112D cell line. Spheroids of this cell line were more resistant than the monolayer when assayed using the PDMS-based microfluidic device, but more sensitive in the Matrigel-assisted ULA plates. It is possible that this cell line is more affected by the Matrigel presence than the others. It is known that ECM components, like Matrigel, can affect drug responses [11]. Although we treated our PDMS devices with pluronic to prevent cell adherence and chemo-absorption [49], performing 3D assays in both PDMS chips and conventional plates enabled us to rule out the well-known absorption of drugs by the hydrophobic porous matrix of the PDMS as a significant cause for this discrepancy [59]. While the absorbed concentration has not been directly controlled, our results (Fig 6B) do not show a systematic increase in resistance in the PDMS devices as would be suggested if the cells are experiencing an effectively lower concentration due to material absorption. Rather, resistance is slightly lower in one cell line [OV866(2)] and significantly higher in another one (TOV112D). Regarding the latter, it is possible that this cell line is more affected by components of the ECM that might influence drug efficacy. Matrigel is a mixture of different ECM components that has high variability issues due to batch effect [60], making it difficult to thoroughly investigate the role of this ECM mimic in the 3D chemosensitivity of this cell line. Therefore, because PDMS-based microfluidic devices were fast, reliable and high-throughput for the analyses of 3D spheroids, and because they used very small media/drug volumes, they make an interesting alternative choice for drug screening either in pharmaceutical research or in clinical settings. In a very recent report, 3D spheroids from tumor tissue of ovarian cancer patients were obtained using the round-bottom ULA plates (without Matrigel) [57]. When compared to clinical outcome, response of these patient-derived 3D spheroids to chemotherapeutic drugs, including the standard of care carboplatin-paclitaxel combination therapy, showed concordance in 89% of the cases (39 of 44) [57]. However, spheroids of different sizes, shapes and compactions were observed from sample to sample and a high number of replicates were needed. The PDMS microfluidic device described here may overcome this technical difficulty by providing a large number of uniform spheroids to be tested for drug response using less patient sample, suggesting that it could easily be incorporated into a clinic setting or used for drug development in the future.

Although descriptive, the present work aims to emit a clear warning that there is no strong tendency of EOC cells to become more resistant in 3D than in 2D, and that spheroid size is an inefficient predictor of cell response as size variation can be both a change of cell number or a change in tissue compactness.

## Conclusion

In drug discovery, the choice of an appropriate *in vitro* pre-clinical model can have a major influence on correctly predicting drug response in human clinical trials. Here, we evaluated carboplatin sensitivity of four epithelial ovarian cancer cell lines using both 2D and 3D *in vitro* models and tested different spheroid forming methods. Channel-based spheroid forming chips held several advantages over commercially available ultra-low attachment plates or the hanging droplet method. Irrespective of the spheroid forming method used, some striking carboplatin sensitivity inversions were noted within a given cell line when 2D and 3D results were compared. Overall, the present study highlights the challenges of *in vitro* models and discusses the different facets of 2D and 3D *in vitro* drug testing.

## Supporting information

S1 Fig 7-AAD and Annexin-V flow cytometry analysis. Graphs represent an example of flow cytometry analysis of spheroids treated with 300 μM carboplatin and its respective control. After excluding cell debris, viable cells were selected based on the absence of 7-AAD and/or Annexin-V markers (red square). Apoptotic and/or dead cells are shown in the other quadrants.
(TIF)

S2 Fig Spheroid formation using hanging drops with different EOC cell lines. (A) Bright field images at day 4. (B) Bright field images at day 6. Scale bar, 500 μm.
(TIF)

S3 Fig Spheroid formation using ULA round-bottom wells (without Matrigel) with different EOC cell lines. Bright field images at day 2. Note that TOV112D cells in these conditions form multiple spheroids of smaller size. Scale bar, 300 μm.
(TIF)
