# Supplementary figures and images for: Carboplatin sensitivity in epithelial ovarian cancer cell lines: The impact of model systems
Source: PLoS One. 2020 Dec 31;15(12):e0244549. doi: 10.1371/journal.pone.0244549 (PMC7774933; doi:10.1371/journal.pone.0244549)

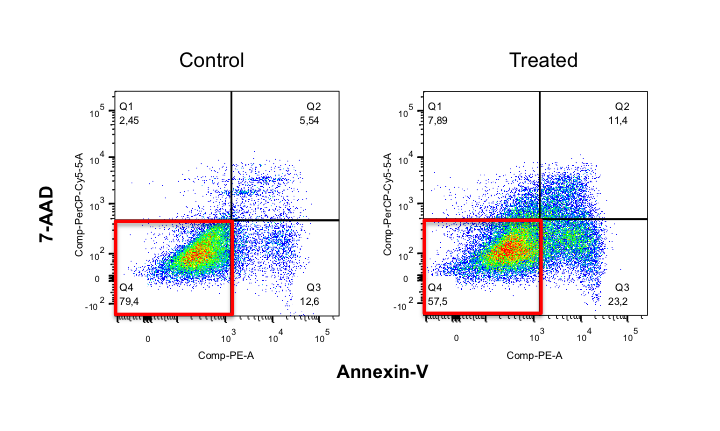

Supplement: S1 Fig — Graphs represent an example of flow cytometry analysis of spheroids treated with 300 μM carboplatin and its respective control. After excluding cell debris, viable cells were selected based on the absence of 7-AAD and/or Annexin-V markers (red square). Apoptotic and/or dead cells are shown in the other quadrants. (TIF) [file pone.0244549.s001.tif]

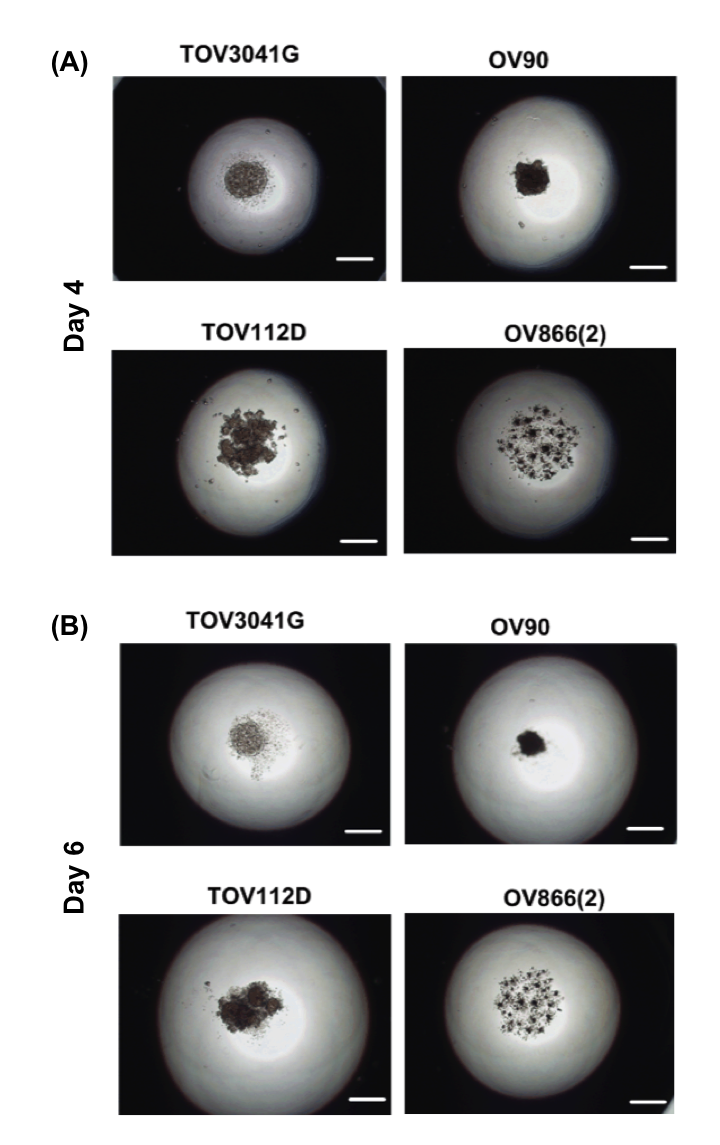

Supplement: S2 Fig — (A) Bright field images at day 4. (B) Bright field images at day 6. Scale bar, 500 μm. (TIF) [file pone.0244549.s002.tif]

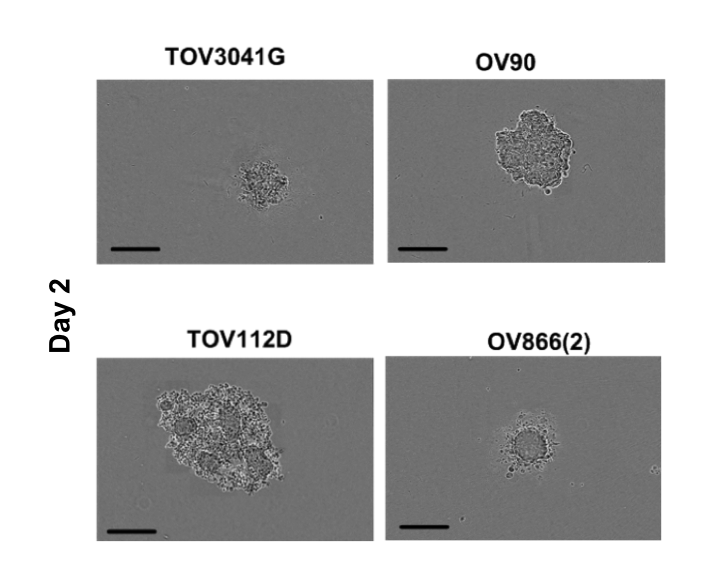

Supplement: S3 Fig — Bright field images at day 2. Note that TOV112D cells in these conditions form multiple spheroids of smaller size. Scale bar, 300 μm. (TIF) [file pone.0244549.s003.tif]
